# Deformation Monitoring of Waste-Rock-Backfilled Mining Gob for Ground Control

**DOI:** 10.3390/s17051044

**Published:** 2017-05-05

**Authors:** Tongbin Zhao, Yubao Zhang, Zhenyu Zhang, Zhanhai Li, Shuqi Ma

**Affiliations:** 1 State Key Laboratory of Mining Disaster Prevention and Control Co-founded by Shandong Province and the Ministry of Science and Technology, Shandong University of Science and Technology, Qingdao 266590, China; ztbwh2001@163.com (T.Z.); zybsdust@163.com (Y.Z.); l_zh2008@163.com (Z.L.); 2 State Key Laboratory of Coal Mine Disaster Dynamics and Control, Chongqing University, Chongqing 400044, China; 3 School of Civil and Environmental Engineering, Nanyang Technological University, 939798 Singapore, Singapore; sqma@ntu.edu.sg

**Keywords:** resistance transducer, deformation, backfilled gob, ground control

## Abstract

Backfill mining is an effective option to mitigate ground subsidence, especially for mining under surface infrastructure, such as buildings, dams, rivers and railways. To evaluate its performance, continual long-term field monitoring of the deformation of backfilled gob is important to satisfy strict public scrutiny. Based on industrial Ethernet, a real-time monitoring system was established to monitor the deformation of waste-rock-backfilled gob at −700 m depth in the Tangshan coal mine, Hebei Province, China. The designed deformation sensors, based on a resistance transducer mechanism, were placed vertically between the roof and floor. Stress sensors were installed above square steel plates that were anchored to the floor strata. Meanwhile, data cables were protected by steel tubes in case of damage. The developed system continually harvested field data for three months. The results show that industrial Ethernet technology can be reliably used for long-term data transmission in complicated underground mining conditions. The monitoring reveals that the roof subsidence of the backfilled gob area can be categorized into four phases. The bearing load of the backfill developed gradually and simultaneously with the deformation of the roof strata, and started to be almost invariable when the mining face passed 97 m.

## 1. Introduction

Solid backfill coal mining is deemed as a scientific and green mining technology, which uses solid waste to backfill the mined-out void (gob) for ground control. This is an effective way to protect underground water resources and surface infrastructure in the vicinity of the mining area [1,2,3]. So far, dry waste rock, cemented waste rock, hydraulic sandfill and paste backfill have been used for backfill in mining. This study focuses on the backfill of dry coal gangue, simultaneously solving the problems of waste rock disposal, minimizing environmental pollution and saving land resources, as typically a large volume of mined-out waste rock is produced with coal resource extraction [4,5,6]. In backfill mining, the movement characteristics of overlying rock strata are distinctly different from traditional cave mining methods. Many studies have been conducted to understand the characteristics of overlying strata movement and ground subsidence of backfill mining. Zhang et al. [7] investigated the characteristics of overburden strata movement by monitoring the support resistance of hydraulic support. Huang et al. [8] developed a thin-plate elastic model to describe the mechanical behavior of overlying strata in backfill mining, and found that the maximum sagging of the immediate roof and main roof decreased with the increase of filling ratios by numerical simulation.

In the process of mining over a certain seam thickness, the overlying rock strata movement and surface subsidence start from the immediate roof, and gradually transfer upward to the ground surface. This significantly threatens the stability of surface infrastructure [9,10,11]. Many numerical studies have been conducted to predict the stress developed in backfilled gob. Fahey et al. [12] found that an arching effect can result in different horizontal stresses along the vertical direction. Mkadmi et al. [13] illustrated that drainage was necessary to reduce pore pressure of backfill, in order to achieve a high level of frictional stress between the backfill and surrounding rock walls. Doherty et al. [14] found that, with cemented paste backfill, the filling and resting schedule plays a dominant role in the development of total stress and pore pressure of backfilled gob. In this study, coal gangue is used, which is mechanically different from paste backfill. Before backfill, blocks of coal gangue were ground into fine granular particles. In order to better understand the whole process of overlying rock strata movement and ground subsidence in backfill mining, field evaluation of the performance of waste-rock-backfilled gob is irreplaceable for optimum backfill design, even though it is costly and time-consuming.

In this study, a real-time deformation monitoring system was firstly developed to monitor the gob roof subsidence and the stress of backfilled material. Based on industrial Ethernet technology, this system contains units of underground data collection, data transmission and surface supervisory. Specific deformation sensors were designed to monitor the roof strata deformation and the stress of backfill material under complicated underground conditions. The developed deformation monitoring system was used to monitor the deformation of waste-rock-backfilled gob in the Tangshan coal mine, Hebei Province, China, and the monitored data was used to analyze the evolution process of the overlying roof strata of the gob.

## 2. Monitoring System Development

### 2.1. Monitoring Scheme

Deformation monitoring covers the displacement of roof subsidence and the stress gradually developed within the backfilled gob. The layout of the monitoring scheme considered the specific geological conditions, the function of monitoring equipment, the precision of monitoring, and the blind spots of monitoring. According to the rule of thumb in monitoring overlying rock strata pressure, the largest subsidence displacement occurs at the roof center of mined-out gob. Therefore, the monitoring station should cover the central area of the gob in the fully-mechanized backfill mining face.

**Figure 1:**
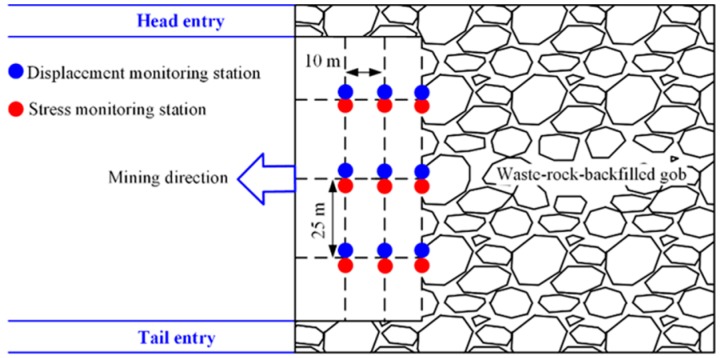
Layout of the gob monitoring scheme.

Three rows of deformation and stress sensors were installed. Figure 1 shows the layout of the monitoring stations for gob roof deformation and stress of the backfill body. In the direction of the advance of the mining face, three monitoring lines were set up with an interval of 10 m between each line. Each monitoring line was then sub-divided into three zones with an interval of 25 m along the direction parallel to the working face. A set of displacement sensors for the gob roof and a set of stress sensors for the backfill body were mounted at every monitoring station.

### 2.2. Online Monitoring System

**Figure 2:**
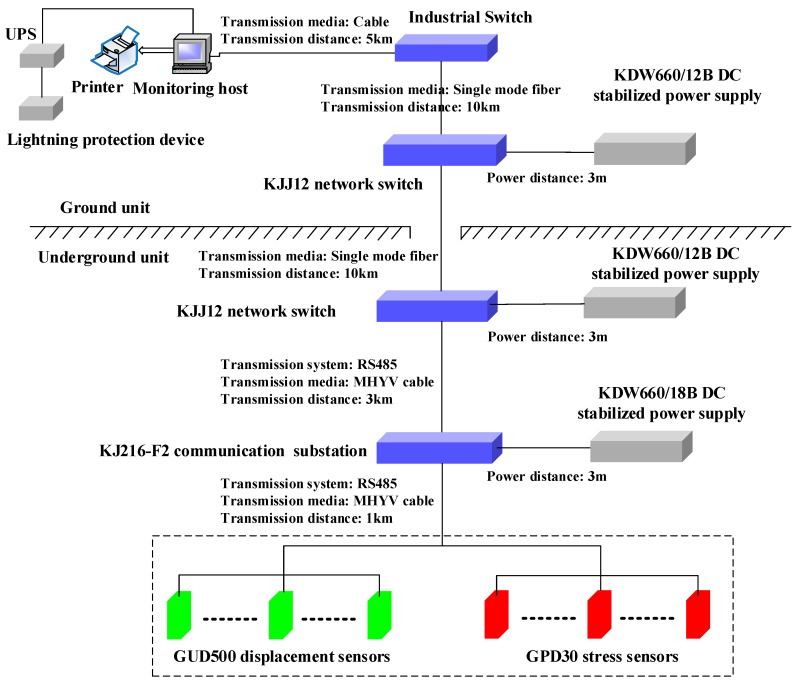
The online deformation monitoring system installed in the backfilled gob.

The online monitoring system contained the ground and underground units, consisting of data acquisition, transmission signal cable, communication monitoring substation, communication monitoring host, and the computer, as shown in Figure 2. The ground unit of the monitoring system was connected with the industrial Ethernet by the monitoring host.

### 2.3. Displacement Sensors

**Figure 3:**
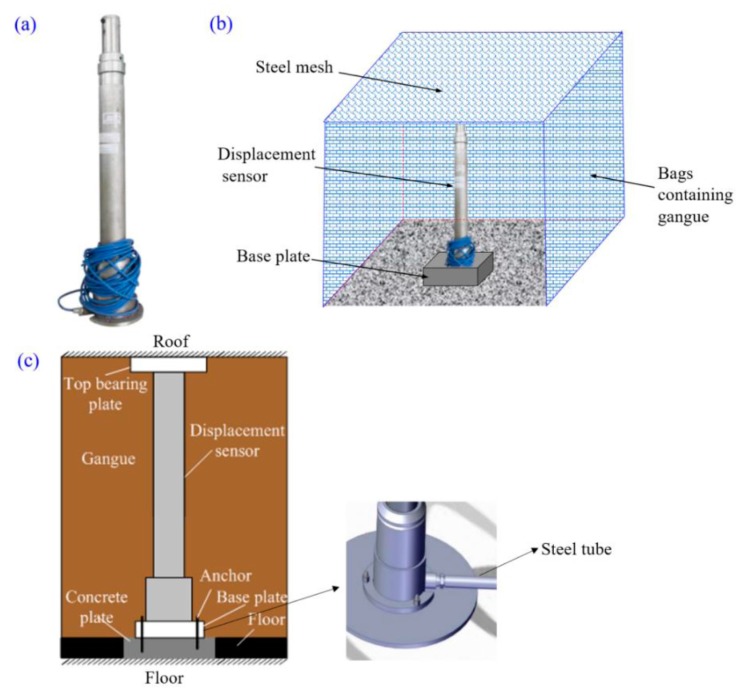
Roof displacement sensor and its installation: (**a**) olumn-type displacement sensor; (**b**) mounting of displacement sensor; (**c**) components of column-typed displacement sensor set-up.

A column-type displacement sensor was used to monitor the roof subsidence of the gob, which employs resistance transducer technology, as shown in Figure 3a. When the backfill body is gradually compacted by the settlement of the roof strata, the inner cylinder assembly of the sensor can be compressed to drive the potentiometer to rotate. By changing the resistance of the precision potentiometer, a linear output voltage signal is generated. The voltage signal is then converted to an RS485 communication signal by the transmitter and communicates with the superior substation. Some performance parameters of the displacement sensor are given in Table 1.

In practice, many difficulties arise in the installation of displacement sensors in a backfilled gob area due to the complicated underground conditions. One challenging issue is that the backfilled waste rock tends to be soft and loose at the early stage of backfill, and it tends to move easily along the horizontal direction once loaded vertically. Such movement of backfill can collapse the deformation sensor. In case of this deformation sensor collapse and data collection failure, the caved-in rock blocks behind the hydraulic support were firstly cleaned up, and then nylon bags filled with waste rock were packed around the column-type displacement sensor to maintain its upright state, as shown in Figure 3b. In order to minimize its negative effect on roof deformation monitoring, the top of the packed nylon bags did not contact the gob roof stratum when setting up the column-type displacement sensor and a certain gap was reserved between them so that the packed nylon bags would not provide any support against roof subsidence.

As shown in Figure 3c, the displacement sensor was vertically positioned in the mined-out gob behind the self-pressure support, and its top and bottom were required to be in tight contact with the immediate roof strata and floor strata of the gob so that the roof deformation could be effectively captured. The bottom of the displacement sensor was fixed on the concrete plate using a ground anchor.

**Figure 4:**
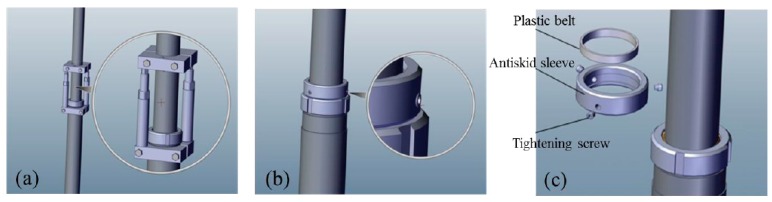
Installation procedures for the displacement sensor: (**a**) spiral push-out device; (**b**) set-up of antiskid sleeve; (**c**). components of antiskid sleeve.

The specific installation procedures of the displacement sensor are as follows: firstly, the waste rock was cleaned up and the internal cylinder of the displacement sensor was pushed out using a spiral push-out device, as shown in Figure 4a. After pushing out the internal cylinder, the three screws of the antiskid sleeve were tightened. The tightening degree was determined by providing the internal cylinder with a resistance of approximately 1500 kN while the outer cylinder does not appear retract, as shown in Figure 4b. The antiskid sleeve cover was equipped a with wear-resistant plastic belt to prevent the tightened screws from making direct contact with the inner surface of the cylinder, as shown in Figure 4c. At last, the displacement sensor was firmly fixed on the base plate with the bolts of M10 × 30, and a cable wire was mounted into the protective steel tube, as shown in Figure 3c.

### 2.4. Stress Sensors

**Figure 5:**
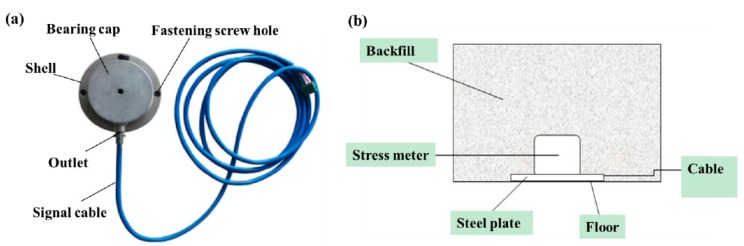
Filling body stress sensor and its installation: (**a**) stress sensor; (**b**) set-up of stress sensor.

The stress sensor was used to measure the vertical stress of the backfilled gob based on the strain measuring technique. The measurement mechanism is activated when, with the advance of the mining face and backfill operation, the roof rock strata gradually subsides to compact the backfill. The vertical stress from the overlying rock strata of the backfilled gob is then transferred to the stress sensor. Such compaction to the stress sensor is finally converted to a voltage signal by means of a strain gauge, and the voltage signal is converted to an RS485 communication signal by the transmitter to communicate with the superior substation. Along with the roof subsidence, the backfill undergoes a dynamic process of compression and the vertical stress gradually develops. As a consequence, Young’s modulus of backfill is a dynamic parameter, which changes with time and space. However, as the roof strata, backfill and stress sensor are continually in contact and there is no separation between any two of them during roof subsidence, the vertical stress within the backfill can therefore be continually transferred from the top to the bottom stress sensor, even though the deformation of the backfill along the vertical direction tends to occur in different gradients due to the difference in Young’s modulus. Therefore, this change in Young’s modulus of backfill has little influence on the stress measurement. Figure 5a shows the components of the stress sensor, and its performance parameters are listed in Table 2.

The stress sensor was installed in backfilled gangue as shown in Figure 5b. Before stress sensor installation, the float coal around the installation station was cleaned and the output signal cables were sequentially labelled. Then, the stress sensor was fixed on the square steel plate with the clamping screw, and the steel plate was fixed to the installation location. The thickness of the steel plate is not less than 5 mm. Finally, the sensor cables were sleeved into a steel conduit or covered by U-shaped steel and gradually connected into the gate road.

## 3. Field Application

### 3.1. Geological Background

**Figure 6:**
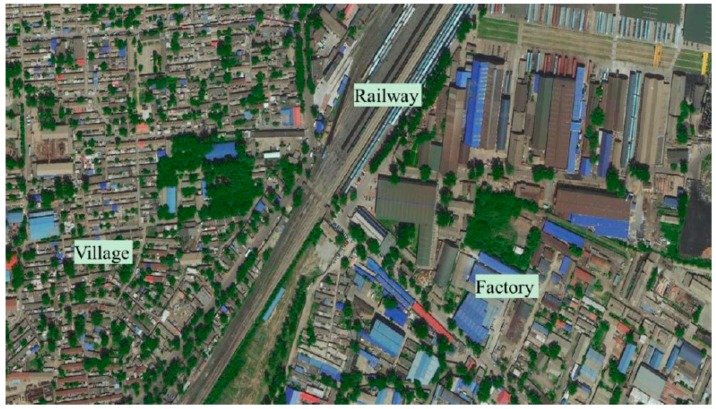
The ground surface buildings of Tangshan coal mine.

The developed online deformation monitoring system was applied to evaluate the ground control performance of the waste-rock-backfilled mining gob in the Tangshan coal mine, Hebei Province, China. Tangshan coal mine has been facing extremely strict public and government scrutiny on ground control, as the coal mine was first founded long ago and subsequently the immediate region has become a metropolitan district due to urbanization. Civic buildings have been gradually constructed on the surface above underground coal seams, as shown in Figure 6. Also, the surface storage of coal gangue is not permitted at the Tangshan mine. The backfilled strip mining method with coal gangue (mined-out waste rock) was introduced to solve the problems of mining-induced ground subsidence and mined-out waste rock. Field monitoring of the deformation of backfilled gob is required in order to evaluate its performance in ground control as well as for the optimization of future backfill operations.

**Figure 7:**
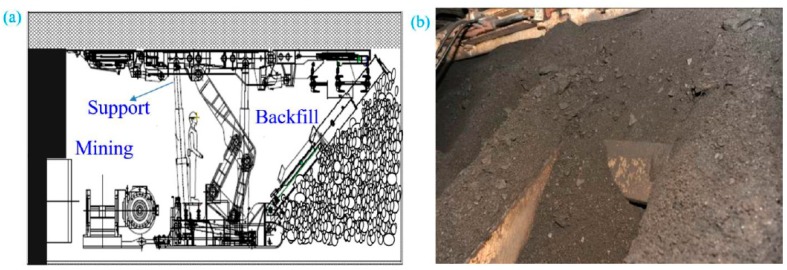
Backfill operation: (**a**) schematic representation of mining and backfill operation, (**b**) backfill photo.

At present, the backfill mining face is the T_3_292 working face, which is located at the 12th level in the north of the Iron District of Tangshan mine. The T_3_292 working face adopts the fully-mechanized strip mining technique with backfill. The coal seam thickness is 4.78 m with the average dipping angle of 12° to the horizon. The mining height is 4.78 m. The mining depth is –700 m. The strike and dip dimensions of T_3_292 longwall mining face are 1170 m and 87 m, respectively. Figure 7 shows the backfill operation in rear of the hydraulic support.

### 3.2. Layout of Monitoring Stations

**Figure 8:**
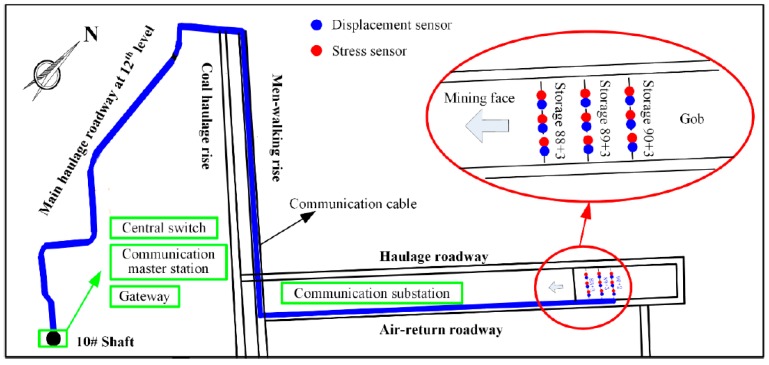
Communication route and the layout of monitoring stations.

**Figure 9:**
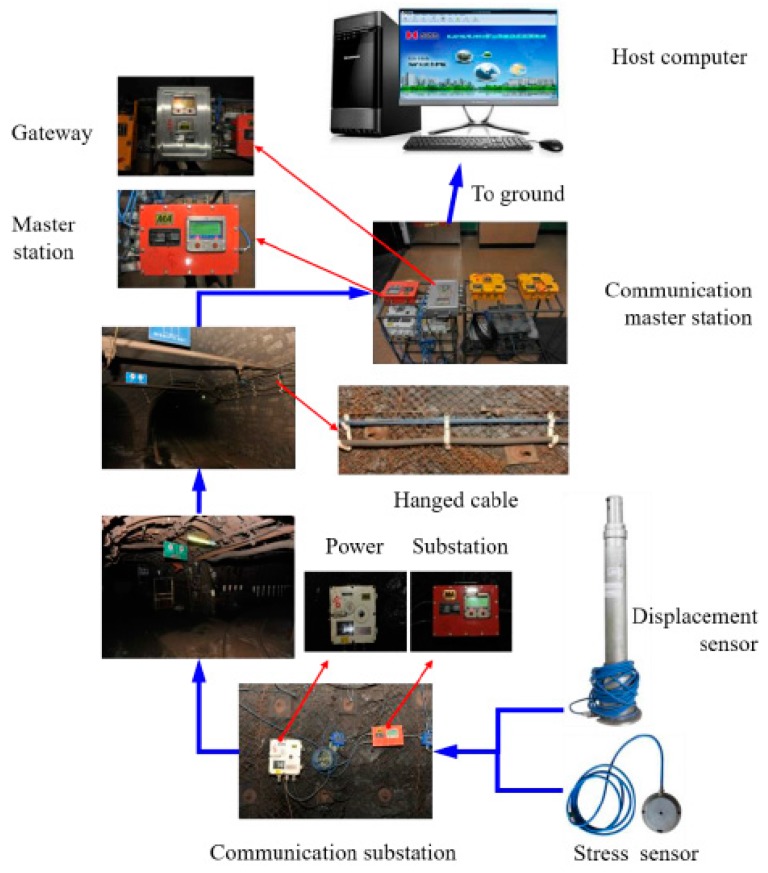
Physical diagram of the field monitoring system.

In the field, the displacement sensors for the overlying rock strata and the stress sensors for the backfilled gob were used to monitor the deformation of the backfilled mining gob. The communication route and the layout of the monitoring stations are schematically shown in Figure 8. The deformation sensors were firstly connected to the communication substation located at the air-return gate road. The communication cable was sequentially laid into the air-return gate road, the walking level, and the main haulage roadway. The total length of the cable extended to 3 km. In a high-voltage power distribution room of the 10# shaft underground opening, as shown in Figure 8, the communication cable was connected to the central switch to the master communication station. Finally, the cable was incorporated to the industrial Ethernet and the collected data was transmitted to the ground monitoring host. The monitoring stations of the backfilled gob were located in Storage 88 + 3, Storage 89 + 3 and Storage 90 + 2, respectively. The data was recorded every 5 s and the pre-warning value of roof subsidence was set to 500 mm for the stability of surface infrastructure. Figure 9 shows the physical set-up of this field monitoring system.

### 3.3. Results and Discussion

**Figure 10:**
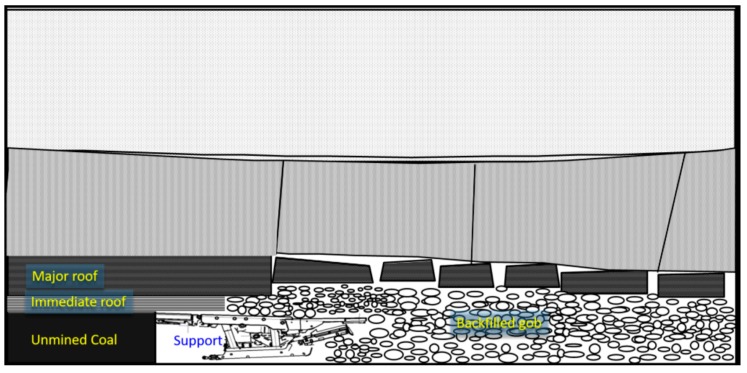
Schematic demonstration of overlying rock strata movement after backfilled mining.

The complexity of the underground backfilled gob environment is the main difficulty for such deformation monitoring. Generally, with the advance of mining, the immediate roof initially cracks and caves in. Subsequently bending or even cracking of the main roof would occur until the backfill is concretely compacted, as schematically shown in Figure 10. Such movement of the overlying roof strata is detrimental to the effectiveness of the deformation monitoring system, as it changes the deformation sensor position and direction, breaks cable lines, etc. In this study, even though nine sensors were installed for both deformation and stress monitoring, only one deformation and one stress sensor successfully monitored the deformation data due to the reasons presented above. 

#### 3.3.1. Displacement Monitoring

**Figure 11:**
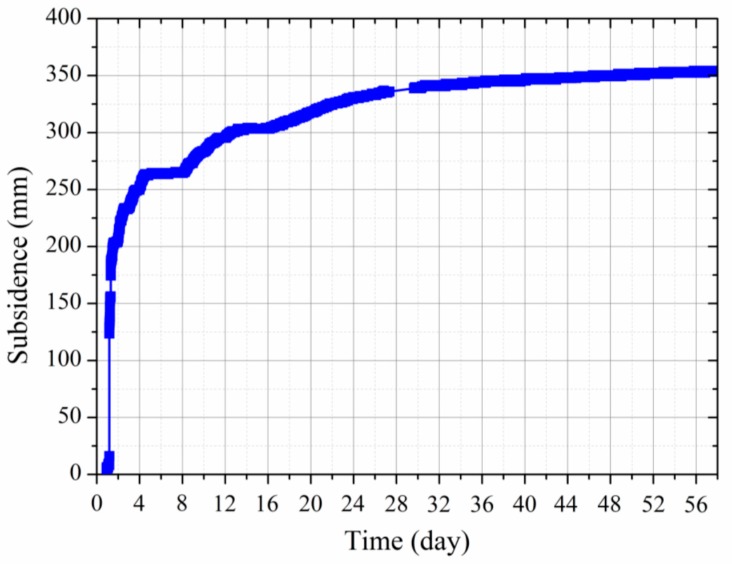
Monitored roof subsidence of backfilled gob over 58 days after backfill.

The normal mining process with the backfill technique involves the process of the mining operation, backfill, maintenance, etc. Therefore, the advance of the mining face does not proceed in a continual time line. Figure 11 shows the monitored roof subsidence with regard to time. It can be seen that, over 58 days after backfill, the monitored maximum roof subsidence of the backfilled gob is 354.2 mm. However, around 74.4% of the total roof subsidence occurred in the first four days and around 22.0% occurred from four to thirty-two days after backfill.

**Figure 12:**
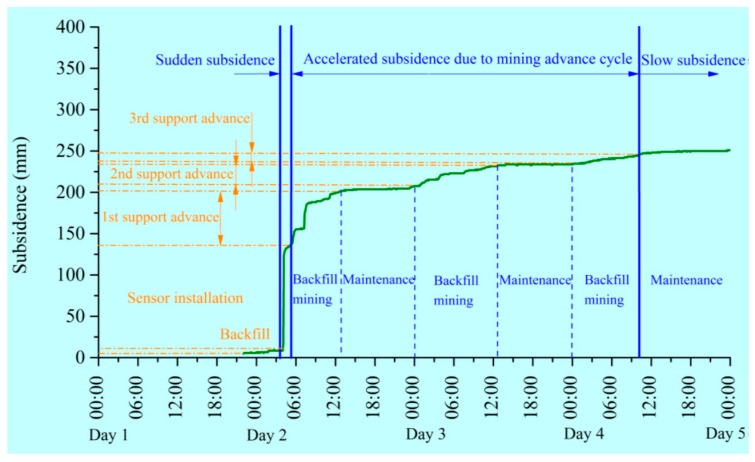
Monitoring the roof subsidence of the backfilled gob at the early stage after backfill.

Figure 12 shows the monitored roof subsidence of the backfilled gob at the early stage of backfill. During the first hydraulic support advance, the roof subsided by 130 mm. After the support contacted the roof strata, the roof continued to subside by 70 mm. During the second and third backfill mining operations, the amount of roof subsidence is 25 mm and 12 mm, respectively. Figure 12 also shows that the amount of roof subsidence during the maintenance periods is relatively small. Therefore, the roof subsidence mainly occurred in the period of support advance. Caution should be taken in these periods in case of roof failure disaster.

**Figure 13:**
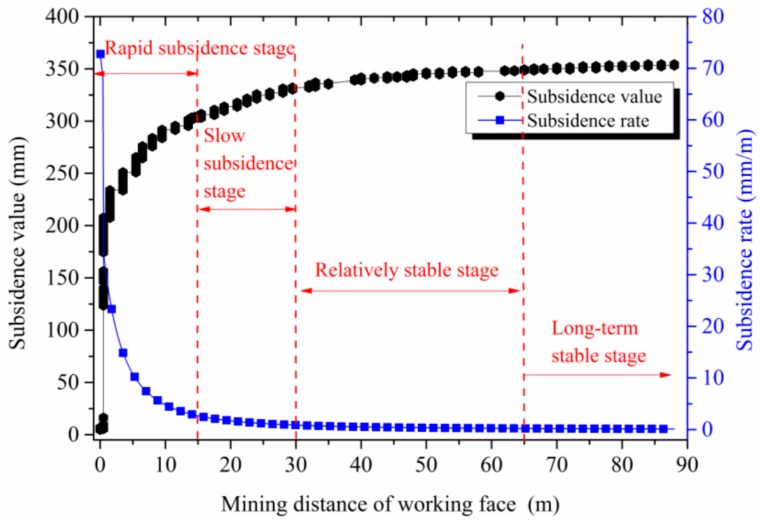
Roof subsidence and roof subsidence rate of the backfilled gob with the advance of the mining face.

Figure 13 shows the cumulative displacement of the roof strata and the rate of roof subsidence with the advance of the mining face. The total displacement of the gob roof subsidence is 353.30 mm. Accordingly, the dynamic evolution of the roof strata deformation can be subdivided into four stages:

(1) Rapid subsidence stage. In this stage, the total roof subsidence of the mined-out gob was 303.30 mm, and the cumulative deformation occurred in the main part of the roof subsidence, approximately 85.8% of the total deformation. This rapid subsidence of the roof was mainly caused by the insufficient contact between the roof and backfilled waste rock, and rapid compaction of the backfill material occurred due to its initial soft nature.
(2) Slow subsidence stage. In this stage, the cumulative roof subsidence was 28.70 mm, approximately 8.1% of the total deformation. During this stage, the roof subsidence rate significantly slowed down. According to statics, it is found that the roof subsidence is dramatically affected by the rate of backfilled mining, and a faster mining speed can produce greater roof subsidence during this stage.
(3) Relatively stable subsidence stage. In this stage, the cumulative roof subsidence was 17.40 mm. This indicates that roof is relatively stable and the influence of mining rate on the roof subsidence is obviously reduced.
(4) Long-term stable stage. In this stage, the cumulative roof subsidence value was only 3.90 mm, approximately 1.1% of the total roof subsidence. The slight subsidence value was mainly caused by the movement of the overlying strata in the gob and was mainly determined by the mechanical properties and developed structure of the overlying rock strata after deformation.

#### 3.3.2. Stress Monitoring

**Figure 14:**
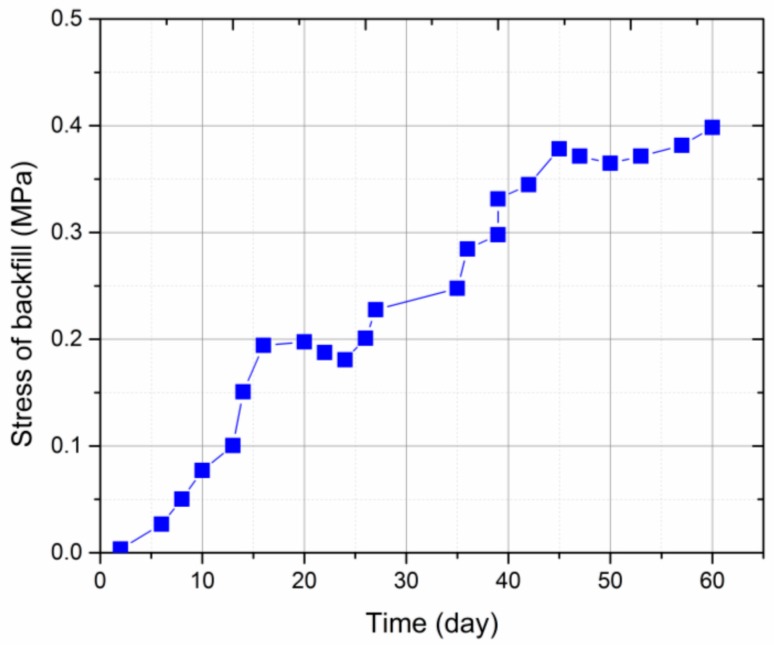
Stress development of the backfill with the time.

The vertical stress development of the backfill body in the gob as a function of time is shown in Figure 14. Over 60 days of stress sensor installation, the vertical bearing load of backfill increased to 0.4 MPa. In the first 13 days after installation, the movement of the overlying rock strata, including roof strata bending, cracking, rotation and cave-in, was violent due to the advance of the mining face and hydraulic support. However, the growth rate of vertical stress in the backfill is not the largest during this period, where it increased with time following a linear trend. Therefore, the vertical stress development of backfill lags behind the movement of the overlying roof strata during this period. This may be because the initial contact between the hydraulic support and backfill is not tight in practical operation. Furthermore, the backfilled waste rock is of a soft and loose nature at the early stage of vertical loading. These characteristics and factors determine such a low growth rate of vertical stress in the backfill. From 13–20 days after sensor installation, the vertical stress in the backfill rapidly increased to 0.2 MPa at a greater growth rate. This is because, with the increase of the gob area due to mining advance, a larger volume of the overlying rock strata has broken and compacted the backfill, which becomes denser and stiffer. Therefore, the overburden load from the roof strata can be effectively transferred to the stress sensor. A slight vertical stress drop occurred from 20–24 days after sensor installation, but this time period was very short. Such a stress drop may be caused by the regional rotation of collapsed large roof blocks, as shown in Figure 10. After that, the vertical stress of the backfill increased again between day 24 and day 44, due to the load transfer by continual subsidence of the overlying roof strata. During the period of day 44 to day 58, the growth of vertical stress of the backfill slowed down, and the final vertical stress tended to stabilize at around 0.4 MPa, indicating that the overlying rock strata tends to be stable with little movement. 

**Figure 15:**
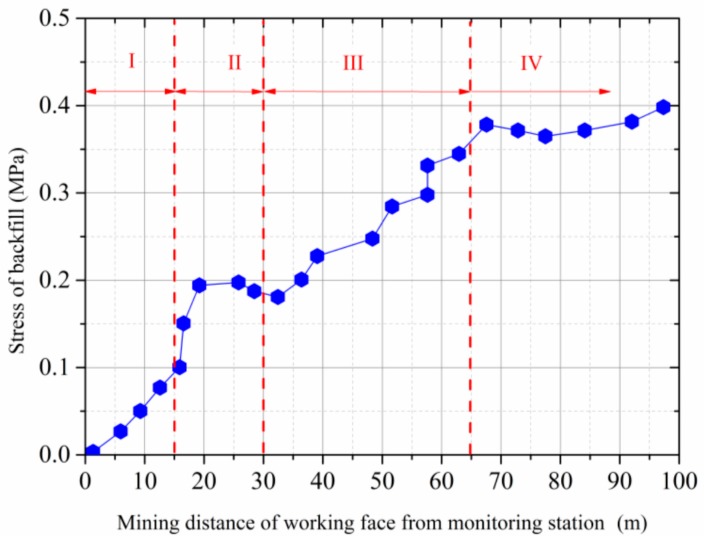
Stress development of the backfill with the advance of the mining face.

Figure 15 shows the stress changes of the backfill with the advance of the mining face. In accordance with the roof deformation, the developed stress of the backfilled material can also be divided into four stages:

(1) Rapid stress development stage I: In this stage, the stress of backfilled rock grew linearly to 0.10 MPa with the advance of the mining face. Generally speaking, the stress development of the backfilled waste rock lags behind the roof subsidence due to the initial loose and soft nature of the backfill material.
(2) Stress regulation stage II: The stress of the backfilled material increased rapidly from 0.10 MPa to 0.20 MPa, then gradually decreased to 0.18 MPa. This fluctuation is caused by the regional cave-in of the roof strata. After the regional cave-in of the roof strata, the pressure of the overlying rock strata could not effectively transfer to the backfill body. Consequently, the overlying strata were co-supported by the backfill and the collapsed roof rock.
(3) Slow stress growth stage III: In this stage, the stress of the backfill slowly increased to 0.38 MPa. With the continual advance of the mining face, the roof strata continued to subside and contact the backfill and collapsed rock again. Therefore, the stress on the sensors started to increase again.
(4) Stress stable stage IV: The stress of the backfill reached the maximum of 0.40 MPa and tended to be stable when the mining face passed the monitoring stations by 97 m. In this stage, the cumulative stress increment of the backfill was only 0.020 MPa, when the mining face advanced by 30 m away, corresponding to the stress growth rate of 0.0067 MPa/m, thus indicating that the overlying rock strata of the backfilled gob tends to be stable.

## 4. Ground Surface Subsidence

The ground surface subsidence above the backfilled gob was also continually monitored to determine the ultimate ground subsidence and evaluate the effect of this backfill mining on surface infrastructure. It was found that the maximum ground surface subsidence is 28 mm. Also, the ground surface subsidence mainly occurred in the first three months after backfill mining. After that, the ground surface tended to be stable, indicating that it is not influenced by the backfill mining any more. Furthermore, no obvious cracks or damage were detected in the surface infrastructure, and the surface infrastructure was still able to be normally used. Therefore, backfilled strip mining in Tangshan Mine can successfully meet the target goal of ground control.

## 5. Conclusions

In this study, an online deformation monitoring system was developed to monitor the deformation of waste-rock-backfilled mining gob. The online monitoring system is composed of a data acquisition sensor, transmission signal cable, communication monitoring substation, communication monitoring host and the computer. The deformation sensors, including roof displacement sensors and stress sensors of the backfill body, were developed employing resistance transducer technology. The deformation sensors and their installation procedure were specifically designed so that they can be used in the complicated underground environment of a mined-out gob. 

The developed monitoring system was practically used to monitor the deformation and stress of waste-rock-backfilled gob at −700 m depth in the Tangshan coal mine, Hebei Province, China. The results show that industrial Ethernet technology can be reliably used for long-term data transmission under complicated underground mining conditions. The monitoring reveals that the roof subsidence of the backfilled gob area can be categorized into four phases, that is, rapid subsidence stage, slow subsidence stage, relatively stable subsidence stage and long-term stable stage. When the mining face passed by the monitoring stations by 90 m, the subsidence of the roof strata reached the maximum value, 353.3 mm. Similarly, the developed stress of the backfill can also be divided into four stages, including the rapid growth stage, the stress regulation stage, the slow growth stage and the stress stable stage. When the mining face passed by the monitoring stations by 97 m, the stress of the backfill increased to its maximum value of 0.40 MPa, and started to be almost invariable.

## Figures and Tables

**Table 1:** Performance parameters of the displacement sensor.

Parameters	Setting
Working voltage	DC 18 V
Working current	≤20 mA
Transmission system	RS485
Cross-sectional area of transmission cables	0.43 mm^2^
Transmission maximum distance	1 km
Measuring span	0–500 mm
Measuring error	≤4.0% (F.S.)
Main dimension	Φ186 × 1200 mm

**Table 2:** Performance parameters of the stress sensor.

Parameters	Setting
Working voltage	DC 18 V
Working current	≤20 mA
Transmission system	RS485
Cross-sectional area of transmission cables	0.43 mm^2^
Transmission maximum distance	1 km
Measuring span	0–30 MPa
Measuring error	≤4.0% (F.S.)
Main dimension	Φ118 × 60.5 mm
